# Predicting the Risk of Rheumatoid Arthritis and Its Age of Onset through Modelling Genetic Risk Variants with Smoking

**DOI:** 10.1371/journal.pgen.1003808

**Published:** 2013-09-19

**Authors:** Ian C. Scott, Seth D. Seegobin, Sophia Steer, Rachael Tan, Paola Forabosco, Anne Hinks, Stephen Eyre, Ann W. Morgan, Anthony G. Wilson, Lynne J. Hocking, Paul Wordsworth, Anne Barton, Jane Worthington, Andrew P. Cope, Cathryn M. Lewis

**Affiliations:** 1 Academic Department of Rheumatology, Centre for Molecular and Cellular Biology of Inflammation, King's College London, London, United Kingdom; 2 Department of Medical and Molecular Genetics, King's College London, London, United Kingdom; 3 Department of Rheumatology, King's College Hospital, London, United Kingdom; 4 Istituto di Genetica delle Popolazioni, Consiglio Nazionale delle Ricerche, Sassari, Italy; 5 Arthritis Research UK Epidemiology Unit, Centre for Musculoskeletal Research, Institute of Inflammation and Repair, The University of Manchester, Manchester, United Kingdom; 6 Division of Musculoskeletal Disease, Leeds Institute of Molecular Medicine, University of Leeds and National Institute for Health Research – Leeds Musculoskeletal Biomedical Research Unit, Leeds, United Kingdom; 7 Academic Unit of Rheumatology, Department of Infection and Immunity, University of Sheffield Medical School, Sheffield, United Kingdom; 8 Musculoskeletal Research Programme, Division of Applied Medicine, University of Aberdeen, Institute of Medical Sciences, Foresterhill, Aberdeen, United Kingdom; 9 NIHR Oxford Musculoskeletal BRU, Nuffield Orthopaedic Centre, Oxford, United Kingdom; 10 Social, Genetic and Developmental Psychiatry Centre (MRC), Institute of Psychiatry, London, United Kingdom; Georgia Institute of Technology, United States of America

## Abstract

The improved characterisation of risk factors for rheumatoid arthritis (RA) suggests they could be combined to identify individuals at increased disease risks in whom preventive strategies may be evaluated. We aimed to develop an RA prediction model capable of generating clinically relevant predictive data and to determine if it better predicted younger onset RA (YORA). Our novel modelling approach combined odds ratios for 15 four-digit/10 two-digit *HLA-DRB1* alleles, 31 single nucleotide polymorphisms (SNPs) and ever-smoking status in males to determine risk using computer simulation and confidence interval based risk categorisation. Only males were evaluated in our models incorporating smoking as ever-smoking is a significant risk factor for RA in men but not women. We developed multiple models to evaluate each risk factor's impact on prediction. Each model's ability to discriminate anti-citrullinated protein antibody (ACPA)-positive RA from controls was evaluated in two cohorts: Wellcome Trust Case Control Consortium (WTCCC: 1,516 cases; 1,647 controls); UK RA Genetics Group Consortium (UKRAGG: 2,623 cases; 1,500 controls). HLA and smoking provided strongest prediction with good discrimination evidenced by an HLA-smoking model area under the curve (AUC) value of 0.813 in both WTCCC and UKRAGG. SNPs provided minimal prediction (AUC 0.660 WTCCC/0.617 UKRAGG). Whilst high individual risks were identified, with some cases having estimated lifetime risks of 86%, only a minority overall had substantially increased odds for RA. High risks from the HLA model were associated with YORA (*P*<0.0001); ever-smoking associated with older onset disease. This latter finding suggests smoking's impact on RA risk manifests later in life. Our modelling demonstrates that combining risk factors provides clinically informative RA prediction; additionally HLA and smoking status can be used to predict the risk of younger and older onset RA, respectively.

## Author Summary

Rheumatoid arthritis (RA) is a common, incurable disease with major individual and health service costs. Preventing its development is therefore an important goal. Being able to predict who will develop RA would allow researchers to look at ways to prevent it. Many factors have been found that increase someone's risk of RA. These are divided into genetic and environmental (such as smoking) factors. The risk of RA associated with each factor has previously been reported. Here, we demonstrate a method that combines these risk factors in a process called “prediction modelling” to estimate someone's lifetime risk of RA. We show that firstly, our prediction models can identify people with very high-risks of RA and secondly, they can be used to identify people at risk of developing RA at a younger age. Although these findings are an important first step towards preventing RA, as only a minority of people tested had substantially increased disease risks our models could not be used to screen the general population. Instead they need testing in people already at risk of RA such as relatives of affected patients. In this context they could identify enough numbers of high-risk people to allow preventive methods to be evaluated.

## Introduction

Rheumatoid arthritis (RA) is a common chronic inflammatory disorder. It results in substantial morbidity and disability alongside high medical and societal costs [1], [2]. There is therefore growing interest in preventing its development. Such prevention requires an ability to reliably predict who will develop RA. Advances in characterising genetic and environmental risk factors for RA together with developments in modelling methodology make predicting its development a realistic possibility.

RA is a clinical syndrome spanning multiple subsets [3]. The commonest subdivision is by the presence or absence of rheumatoid factor (RF)/anti-citrullinated protein antibodies (ACPA), termed seropositive and seronegative RA respectively. Risk factor evaluation has mainly focussed on seropositive RA with nearly half its genetic architecture known. *HLA-DRB1* alleles, in particular those encoding the shared epitope, dominate genetic risk accounting for approximately 36% of heritability [4]; 45 non-HLA variants explain approximately 15% of heritability [4]. Smoking is the main environmental risk factor [5]; it predisposes to seropositive RA and has a synergistic relationship with the shared epitope [6], [7]. Although single factors do not provide sufficient risk stratification, combining multiple factors within a prediction model may identify clinically relevant high- and low-risk groups. The large risks conferred by HLA make such modelling an attractive prospect in RA despite limited success in other complex disorders [8]–[10].

RA develops over many years prior to clinical presentation [11]. Initially, individuals with genetic susceptibility variants are exposed to environmental risks; some may develop autoantibodies (RF/ACPA) [12]. A proportion will subsequently develop arthralgia, which may progress to an unclassified arthritis followed by a fully expressed RA phenotype. Pilot studies in unclassified arthritis indicate that secondary prevention may be possible with corticosteroids [13], [14], methotrexate [15] and biologics [16] attenuating the progression to RA. Although preventive treatments may be more effective before immune dysregulation and symptoms develop, primary prevention is not currently possible as no reliable method exists to identify asymptomatic high-risk individuals.

Prevention is likely to have a larger impact in younger onset RA (YORA) due to the increased health costs associated with a longer disease duration [17]. Genetic susceptibility factors may influence RA's age of onset with HLA-DRB1*04 alleles [18]–[21] and multiple single nucleotide polymorphisms (SNPs) such as those tagging *VEGFA* [22], *RANKL* [19], [23], *MMP1-3* [22] and *PTPN22* [24], [25] loci associating with YORA.

One group has published two reports outlining predictive models for RA. Their models, built using 8 HLA alleles, 14–31 SNPs and clinical factors, generated an aggregate weighted genetic risk score (wGRS) formed from the product of individual-locus odds ratios (ORs) [26], [27]. They were reasonably accurate at determining disease status in approximately 1,200 cases and 1,200 controls, with a maximal area under the curve (AUC) of 0.752. They also demonstrated a better ability to predict erosive RA (a more severe phenotype). However, only a minority of the studied populations had significantly elevated risks for RA.

We report an alternative modelling approach to predicting RA. Our novel modelling method uses computer simulation to categorise risk profiles; our models also incorporate a larger number of HLA risk variants. The risk factors included in our modelling comprise 15 four-digit/10 two-digit *HLA-DRB1* alleles, 31 SNPs and male ever-smoking status (as ever-smoking is a significant risk for RA in males only). We applied our models to two large cohorts of European ancestry: the Wellcome Trust Case Control Consortium (WTCCC) and the UK RA Genetics Group (UKRAGG) Consortium. Our primary aim was to determine if our approach would generate clinically relevant predictive values. Our secondary aim was to determine if our modelling better identified YORA. We demonstrate that clinically informative RA risk prediction is possible and that the risk of younger and older onset RA can be predicted using information on HLA and smoking status, respectively.

## Materials and Methods

### Ethics Statement

All participants in WTCCC and UKRAGG were recruited after providing informed consent. UKRAGG was approved by the North West Multi-Centre Research Ethics Committee (MREC 99/8/84). Authors gained written permission and approval from WTCCC to undertake this work in the publically available WTCCC1 collections.

### Study Populations

The WTCCC dataset contains SNP data on 1,999 RA cases and 3,004 controls [28]. Controls were obtained from the 1958 British Birth Cohort and UK Blood Services. Genotyping was performed on the Affymetrix GeneChip 500k Mapping Array Set. Quality control (QC) procedures were undertaken excluding individuals with <97% SNP call rates, high heterozygosity, non-European ancestry or relatedness, discordance between genotype and phenotype data and duplicate samples. In the post-QC dataset information was available on 490,031 SNP markers; the total genotyping rate was 1.00. Two- or four-digit resolution *HLA-DRB1* tissue typing data were available on 1,837 cases and 1,647 controls.

The UKRAGG dataset contains SNP data on 5,024 RA cases and 4,281 controls from 6 UK centres [29]. Genotyping was performed using the Sequenom platform. Four hundred and four SNPs were genotyped over 8 staggered plexes; for each plex separate QC was undertaken excluding individuals and SNPs with <90% data present. In the post-QC dataset total genotyping rates were 0.73 owing to systematic differences in samples run on each plex. Two- or four-digit resolution *HLA-DRB1* tissue typing data were available on 3,420 cases and 1,500 controls.

Both datasets contained cases fulfilling the 1987 ACR classification criteria for RA [30]. *HLA-DRB1* tissue typing was undertaken (at two-digit or four-digit resolution) at individual centres, using commercially available semiautomated polymerase chain reaction-sequence-specific oligonucleotide probe (PCR-SSOP) typing techniques (or research assays based on PCR-SSOP linear array technology) [29]. Two-digit typing includes the allele group (Field 1) only; four-digit typing includes both the allele group and the allele subtype encoding a specific HLA protein (Field 2) (http://hla.alleles.org/nomenclature/naming.html).

We undertook prediction modelling in seropositive cases and controls with *HLA-DRB1* tissue typing data available with or without additional SNP and smoking data (as most replicated risk loci are for seropositive RA and genetic risk is dominated by HLA) [4], [31]. The final cohorts comprised 1,516 cases and 1,647 controls from WTCCC and 2,623 cases and 1,500 controls from UKRAGG (Table 1).

**Table 1:** Clinical characteristics of WTCCC/UKRAGG cases and controls included in modelling.

		WTCCC		UKRAGG	
		RA (n = 1,516)	Controls (n = 1,647)	RA (n = 2,623)	Controls (n = 1,500)
*Gender*	Female	1,151 (76.0)	739 (50.0)	1,868 (71.2)	890 (59.9)
*RA Characteristics*	RF+	1,452 (96.1)	-	2,385 (93.1)	-
	ACPA+	1,061 (86.5)	-	1,508 (84.8)	-
	Mean Age Of Onset (95% CI)	45.3 (44.6–46.1)	-	48.0 (47.5–48.6)	-
	Erosive Disease	1,009 (71.1)	-	830 (69.7)	-
	Nodules	-	-	859 (38.3)	-
*Smoking Status*	Male Ever-Smokers	231 (80.5)^a^	422 (57.1)^a^	417 (78.8)^a^	149 (46.3)^a^
	Female Ever-Smokers	552 (58.3)^b^	425 (57.7)^b^	758 (55.9)^b^	238 (39.3)^b^

Data are number (%) unless otherwise stated. The following data are missing from WTCCC: gender in 2 cases and 169 controls; RF status in 5 cases; ACPA status in 290 cases; age of onset missing/inaccurate in 63 cases; erosive status in 96 cases; smoking status in 76 male cases, 204 female cases and 3 female controls. The following data are missing from UKRAGG: gender in 14 controls; RF status in 60 cases; ACPA status in 844 cases; age of onset missing/inaccurate in 93 cases; erosive status in 1,432 cases; nodular status in 378 cases; smoking status in 226 male cases, 513 female cases, 274 male controls and 284 female controls.

a = % of males that are ever smokers;

b = % of females that are ever smokers.

### Prediction Modelling Overview

Our modelling was performed within the R package, REGENT (Risk Estimation for Genetic and Environmental Traits), developed within our unit. This program incorporates published gene-environment risk factor and disease statistics to categorise risk using a confidence interval (CI)-based approach within a simulated population. The methodology underlying REGENT has previously been described in detail [32], [33].

Genetic and environmental risk factors for input into REGENT are selected from the literature. Genetic risk factors require allelic ORs, allele frequencies, and sample sizes from relevant studies, in order to estimate precision. Environmental risk factors require ORs, standard errors and the proportion of the population exposed to the risk factor. Data on these risk factors are entered into REGENT as summary statistic input files, which are processed in two stages: the first develops the prediction model and the second runs the prediction model in real life data.

In the first stage REGENT simulates a population-distribution of disease risk. Risk profiles are simulated based on the frequency of each risk factor in the general population. Summary ORs for each risk profile are generated through combining the ORs for each genetic and environmental risk factor in a multiplicative model that assumes risk factor independence. CIs are generated using information on the variability of genetic risk factors (derived from the sample size of the risk variant discovery cohort) and environmental risk factors (standard error of the effect size). Each simulated risk profile's OR is initially calculated relative to a profile with no risk factors present; these are subsequently adjusted to ensure correct disease prevalence in the population, assigning a risk profile with a mean OR as having a baseline risk of 1.0. CIs are used to classify risk profiles into four risk categories (reduced, average, elevated and high-risk). Starting with the risk profile of baseline risk (OR = 1.0), any risk profile whose CI overlaps with this baseline CI is classified as being of average-risk (as this profile is not statistically different from baseline). Any risk profile whose CI resides fully below the baseline CI is classified as reduced-risk. Profiles with CIs above the baseline CI are classified as elevated-risk. Furthermore, a high-risk group is determined by profiles whose CIs reside completely above the CI of the first risk profile classified as elevated-risk. An example of how this process is undertaken in a simplified model using 3 SNPs is provided in Figure S1.

In the second stage REGENT applies this simulated population profile to individual level data. Genotypes and environmental risk factor exposure data on each individual in the dataset of interest (WTCCC and UKRAGG) are entered into REGENT, which generates two measures of disease risk. Firstly, each individual's summary OR (95% CI) for RA is calculated (relative to the baseline individual with an OR of 1.0); as with the simulated population, risk factors are combined in a multiplicative model. This summary OR informs the individual of their risk of developing RA. Secondly, each individual is assigned a risk category for RA. This is undertaken through comparing the CI of each individual's summary OR to those of the simulated risk distribution in the same manner as described in stage 1. This risk category informs an individual whether they are at an increased or reduced risk of disease, relative to the average person in the general population.

### Prediction Model Components Identified from Meta-Analyses

#### Genetic Risk Factors

We identified genetic susceptibility variants for potential inclusion in our prediction modelling from two large, recently published meta-analyses [34], [35]. We sought to include only susceptibility alleles attaining genome-wide significance (P_GWAS_<5×10^−8^); this ensured that the alleles modelled were replicated RA genetic risk factors. These comprised 15 four-digit and 10 two-digit *HLA-DRB1* alleles and 35 non-HLA SNPs.

#### Environmental Risk Factor

We included the environmental risk factor smoking in our modelling. Other factors proposed to influence RA risk such as alcohol were not included: firstly the evidence underlying these is uncertain, with associations often present in case-control and not cohort studies [36] and secondly detailed data on non-smoking risk factors were not captured in WTCCC and UKRAGG.

We used published ORs from the most recent meta-analysis evaluating smoking as an RA risk factor [5]. In this meta-analysis ever-smoking was a significant risk for seropositive RA in males only (OR 3.02; 95% CI 2.35–3.88) with a substantially smaller and non-significant (CIs contain 1) impact seen in females (OR 1.34; 95% CI 0.99–1.80). We therefore hypothesized that smoking would not improve prediction in women (confirmed in preliminary analyses; Table S1). As a result only males were evaluated in our modelling incorporating ever-smoking.

Although smoking interacts with the shared epitope we did not factor this into our modelling. This is because studies reporting summary ORs for this interaction [6], [29], [37], [38] have marked heterogeneity between them; therefore using meta-analysis techniques to obtain pooled ORs for shared epitope-smoking status combinations would be inaccurate and thus inappropriate. Examples of this heterogeneity include: (1) studies reporting risks stratified by different smoking levels, which would require an inverse variance fixed-effects model to obtain common ORs for all smokers within studies in addition to a random-effects model to estimate pooled ORs across studies; (2) two studies classifying the shared epitope at two-digit resolution, thus incorporating non-shared epitope alleles [6], [37]; (3) two studies not including all known shared epitope alleles [29], [38].

### Prediction Model Component Availability in WTCCC and UKRAGG

Two-digit or four-digit *HLA-DRB1* tissue typing data were available in all evaluated individuals. In WTCCC 1,342 seropositive cases, 966 ACPA-positive cases and 1,126 controls had four-digit resolution data available on both alleles; 29 seropositive cases, 14 ACPA-positive cases and 159 controls had two-digit resolution data available on both alleles; 145 seropositive cases, 81 ACPA-positive cases and 362 controls had mixed-digit resolution data (one *HLA-DRB1* allele known at four-digit and the other at two-digit resolution) available. In UKRAGG 1,534 seropositive cases, 1,108 ACPA-positive cases and 735 controls had four-digit resolution data available on both alleles; 312 seropositive cases, 66 ACPA-positive cases and 205 controls had two-digit resolution data available on both alleles; 777 seropositive cases, 334 ACPA-positive cases and 560 controls had mixed-digit resolution data available.

We excluded 4 SNPs attaining P_GWAS_ in the meta-analysis for the following reasons: 1 (rs11676922) was in high linkage disequilibrium (r^2^>0.9; HapMap release 22 CEU population panel) [39] with another (rs10865035) – in this case the latter SNP was included due to a previous association with RA – and 3 SNPs/proxy SNPs were unavailable (rs10488631, rs6859219 and rs934734 in UKRAGG; rs6822844, rs874040 and rs951005 in WTCCC). Eleven and two proxy SNPs were used in WTCCC and UKRAGG respectively (Table S2) [39].

Data on ever-smoking status were available in 287 male cases and 739 male controls in WTCCC and 529 male cases and 322 male controls in UKRAGG.

### Final Prediction Models

To examine the contribution of each gene-environment component to prediction we constructed several models. These comprised a SNP model (with 31 SNPs), an HLA model (10 two-digit and 15 four-digit *HLA-DRB1* alleles), an HLA-SNP model (combining HLA and SNP model components), an HLA-smoking model (combining *HLA-DRB1* alleles with ever-smoking status) and an HLA-SNP-smoking model (combining *HLA-DRB1* alleles, 28 SNPs and ever-smoking status). Only the 28 SNPs present in both WTCCC and UKRAGG were incorporated in the last model. The latter two models, which included smoking, were evaluated in males only.

The decision to combine two-digit and four-digit *HLA-DRB1* alleles in the HLA model was undertaken to avoid removing the substantial number of individuals with mixed resolution typing. Preliminary analyses confirmed the validity of this approach with no significant differences seen in the discriminative abilities of HLA models incorporating (1) two-digit alleles only; (2) four-digit alleles only and (3) a mixed resolution of alleles (Table S3). Within our mixed resolution modelling the risks for each HLA allele were included only once per individual at the highest resolution at which they were known.

**Figure 1:**
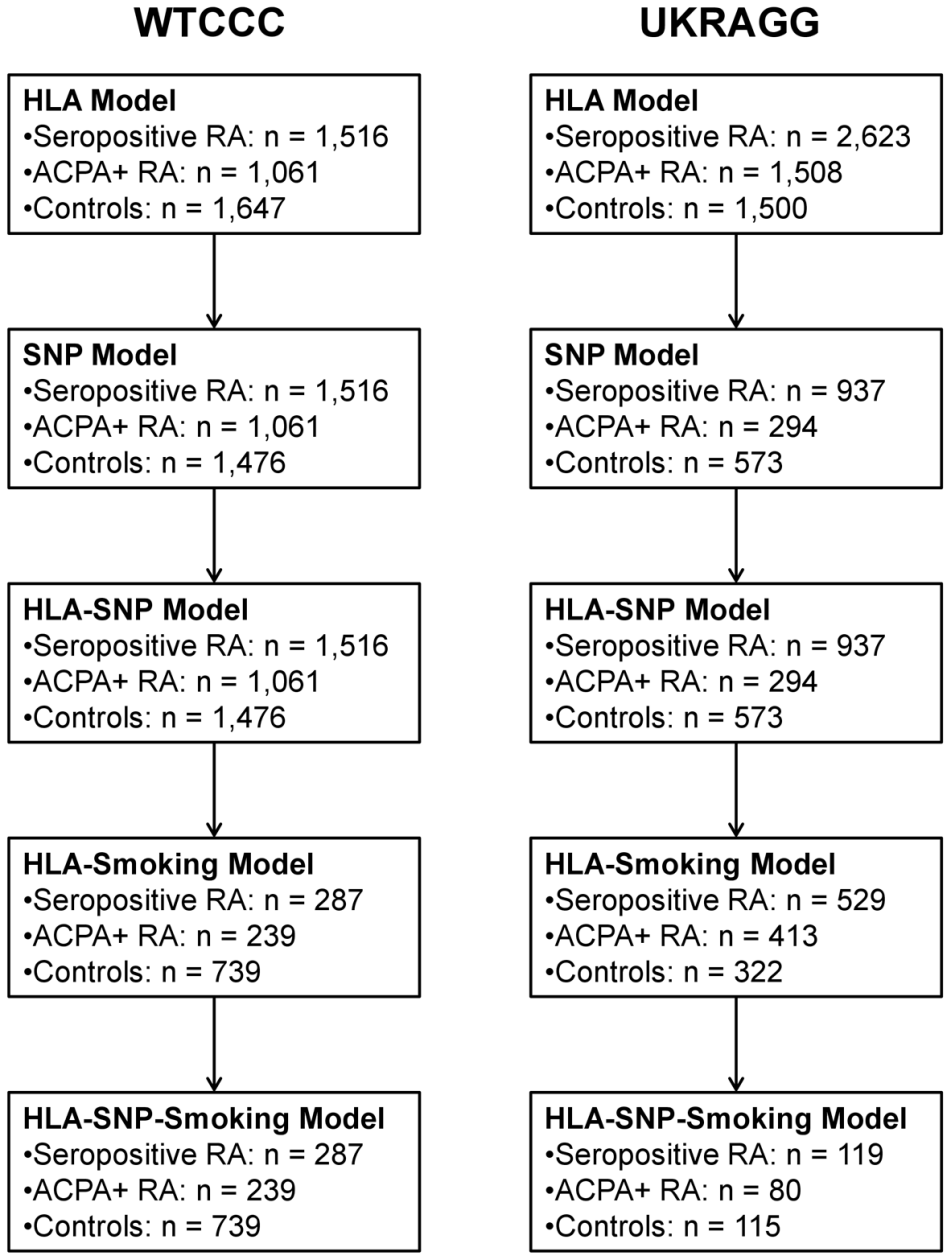
Number of individuals evaluated in each prediction model.

Only individuals with available data on relevant risk factors were included in models incorporating those risk factors. Therefore only males with available smoking data were included in the HLA-smoking and HLA-SNP-smoking models. Similarly only individuals with data available on the modelled SNPs could be included in the HLA-SNP and HLA-SNP-smoking models. Owing to missing data the number of individuals evaluated in each prediction model fell as more risk factors were included (Figure 1).

### Statistical Analyses

#### Evaluating Dataset Validity

To compare the representativeness of our datasets to published RA populations we summarised clinical features of cases and controls (Table 1) and calculated effect allele frequencies and allelic ORs (95% CIs) (Tables 2 and 3). For the *HLA-DRB1* allele case-control association analysis (Table 2) the two-digit resolution allele results included both individuals with two-digit resolution typing and collapsed four-digit resolution typing. This approach was undertaken due to the small number of individuals with two-digit typing data in WTCCC/UKRAGG. The meta-analysis from which we obtained our risk alleles had almost identical allele frequencies when comparing two-digit alleles and four-digit alleles collapsed to two-digit resolution [35]; comparing our datasets to the meta-analysis findings in this manner was therefore appropriate.

**Table 2:** Classical *HLA-DRB1* allele frequencies and their association with seropositive RA in WTCCC and UKRAGG.

	Published Meta-Analysis [35]			WTCCC			UKRAGG		
*HLA-DRB1* Allele	OR (95% CI)	MAF Co	MAF Ca	OR (95% CI)	MAF Co	MAF Ca	OR (95% CI)	MAF Co	MAF Ca
*01	1.30 (1.21–1.40)	0.113	0.145	1.53 (1.31–1.78)	0.104	0.151	1.27 (1.11–1.45)	0.121	0.149
*01:01	1.38 (1.28–1.50)	0.097	0.133	5.88 (4.62–7.55)	0.026	0.136	1.25 (1.06–1.47)	0.081	0.099
*03	0.59 (0.54–0.64)	0.128	0.082	0.67 (0.58–0.78)	0.148	0.105	0.76 (0.67–0.86)	0.159	0.125
*03:01	0.59 (0.54–0.64)	0.128	0.082	0.65 (0.55–0.76)	0.145	0.099	0.44 (0.37–0.51)	0.130	0.061
*04	3.71 (3.49–3.93)	0.174	0.450	2.90 (2.59–3.24)	0.213	0.439	3.19 (2.86–3.56)	0.184	0.419
*04:01	4.14 (3.86–4.44)	0.104	0.309	2.93 (2.57–3.35)	0.124	0.293	3.00 (2.63–3.42)	0.111	0.272
*04:04	3.17 (2.83–3.54)	0.036	0.091	1.86 (1.52–2.28)	0.052	0.092	2.56 (2.08–3.18)	0.039	0.093
*04:05	2.31 (1.77–3.01)	0.007	0.012	2.01 (1.12–3.73)	0.006	0.012	2.61 (1.34–5.58)	0.004	0.010
*04:08	5.48 (4.11–7.30)	0.005	0.017	-a	0.000	0.021	2.78 (1.70–4.76)	0.007	0.018
*07	0.49 (0.45–0.54)	0.133	0.064	0.48 (0.41–0.56)	0.154	0.080	0.54 (0.46–0.62)	0.142	0.081
*07:01	0.49 (0.45–0.54)	0.133	0.064	0.41 (0.35–0.49)	0.154	0.070	0.37 (0.32–0.44)	0.140	0.057
*08	0.41 (0.34–0.50)	0.029	0.013	0.39 (0.24–0.62)	0.022	0.009	0.30 (0.20–0.44)	0.029	0.009
*08:01	0.34 (0.26–0.44)	0.019	0.009	0.27 (0.13–0.53)	0.014	0.004	0.69 (0.33–1.46)	0.005	0.003
*10	2.53 (2.04–3.14)	0.008	0.020	1.97 (1.11–3.59)	0.006	0.012	1.75 (1.04–3.07)	0.007	0.012
*10:01	2.53 (2.04–3.14)	0.008	0.020	1.97 (1.11–3.59)	0.006	0.012	1.48 (0.85–2.67)	0.006	0.009
*11	0.48 (0.43–0.54)	0.094	0.039	0.50 (0.39–0.64)	0.064	0.033	0.42 (0.34–0.53)	0.065	0.028
*11:01	0.44 (0.38–0.52)	0.061	0.028	0.80 (0.55–1.14)	0.023	0.018	0.33 (0.23–0.47)	0.030	0.010
*11:04	0.15 (0.10–0.23)	0.024	0.008	0.79 (0.41–1.49)	0.008	0.006	0.38 (0.15–0.91)	0.005	0.002
*13	0.33 (0.30–0.37)	0.114	0.044	0.41 (0.33–0.50)	0.098	0.042	0.46 (0.38–0.55)	0.084	0.040
*13:01	0.28 (0.24–0.33)	0.061	0.021	0.77 (0.54–1.08)	0.026	0.020	0.42 (0.29–0.59)	0.027	0.011
*13:02	0.29 (0.23–0.38)	0.027	0.012	0.59 (0.38–0.90)	0.020	0.012	0.27 (0.18–0.42)	0.023	0.006
*14	0.50 (0.40–0.62)	0.025	0.012	0.51 (0.34–0.76)	0.024	0.013	0.45 (0.31–0.65)	0.023	0.010
*14:01	0.46 (0.36–0.59)	0.022	0.011	0.43 (0.28–0.66)	0.024	0.011	0.67 (0.34–1.33)	0.006	0.004
*15	0.59 (0.54–0.64)	0.142	0.092	0.63 (0.53–0.75)	0.128	0.084	0.60 (0.53–0.70)	0.146	0.093
*15:01	0.57 (0.53–0.62)	0.136	0.089	1.09 (0.87–1.37)	0.051	0.055	-a	0.000	0.025

All alleles attained genome-wide significance in the published meta-analysis; MAF = minor allele frequency; Co = controls; Ca = Cases;

a = OR incalculable due to no allele copies in the control group.

**Table 3:** Non-HLA RA susceptibility SNP allele frequencies and their association with seropositive RA in WTCCC and UKRAGG.

	Published Meta-Analysis [34]			WTCCC		UKRAGG	
Loci	SNP	MAF^a^	OR	MAF Ca/Co	OR (95% CI)	MAF Ca/Co	OR (95% CI)
*PTPN22*	rs2476601	0.10	1.94 (1.81–2.08)	0.18/0.10	2.02 (1.73–2.36)	0.16/0.10	1.60 (1.38–1.85)
*TNFAIP3*	rs6920220	0.22	1.22 (1.16–1.29)	0.27/0.23	1.26 (1.12–1.41)	0.25/0.21	1.29 (1.15–1.44)
*ANKRD55, IL6ST*	rs6859219	0.21	0.78 (0.72–0.85)	0.17/0.20	0.80 (0.70–0.91)	-	-
*CD40*	rs4810485	0.25	0.85 (0.80–0.90)	0.22/0.24	0.87 (0.77–0.99)	0.22/0.25	0.83 (0.74–0.93)
*CTLA4*	rs3087243	0.44	0.87 (0.83–0.91)	0.43/0.44	0.95 (0.86–1.06)	0.43/0.47	0.86 (0.78–0.94)
*TNFAIP3*	rs5029937	0.04	1.40 (1.24–1.58)	0.06/0.04	1.58 (1.24–2.02)	0.05/0.04	1.39 (1.06–1.82)
*IL2RA*	rs706778	0.40	1.14 (1.09–1.20)	0.46/0.42	1.17 (1.05–1.29)	0.43/0.40	1.13 (1.02–1.25)
*RBPJ*	rs874040	0.30	1.14 (1.08–1.20)	-	-	0.33/0.31	1.11 (1.00–1.23)
*TRAF1, C5*	rs3761847	0.43	1.13 (1.08–1.18)	0.45/0.46	0.96 (0.87–1.07)	0.46/0.43	1.12 (1.01–1.24)
*STAT4*	rs7574865	0.22	1.16 (1.10–1.23)	0.21/0.19	1.12 (0.99–1.27)	0.25/0.22	1.18 (1.05–1.32)
*SPRED2*	rs934734	0.49	1.13 (1.08–1.19)	0.53/0.51	1.11 (1.00–1.23)	-	-
*CCR6*	rs3093023	0.43	1.13 (1.08–1.19)	0.42/0.40	1.10 (0.99–1.22)	0.47/0.44	1.16 (1.05–1.28)
*PXK*	rs13315591	0.09	1.29 (1.17–1.43)	0.10/0.09	1.11 (0.94–1.32)	0.08/0.07	1.10 (0.91–1.33)
*C5orf30*	rs26232	0.32	0.88 (0.84–0.93)	0.34/0.40	0.78 (0.70–0.86)	0.31/0.31	1.03 (0.92–1.14)
*CCL21*	rs951005	0.16	0.84 (0.78–0.90)	-	-	0.13/0.15	0.86 (0.75–0.99)
*REL*	rs13031237	0.37	1.13 (1.07–1.18)	0.45/0.43	1.07 (0.96–1.18)	0.41/0.37	1.22 (1.10–1.35)
*AFF3*	rs10865035	0.47	1.12 (1.07–1.17)	0.50/0.46	1.19 (1.07–1.31)	0.48/0.45	1.16 (1.05–1.27)
*PRKCQ*	rs4750316	0.19	0.87 (0.82–0.92)	0.16/0.20	0.77 (0.67–0.87)	0.18/0.19	0.89 (0.79–1.00)
*IRF5*	rs10488631	0.11	1.19 (1.10–1.28)	0.12/0.10	1.22 (1.04–1.44)	-	-
*TNFRSF14*	rs3890745	0.32	0.89 (0.85–0.94)	0.29/0.32	0.85 (0.76–0.95)	0.32/0.33	0.97 (0.88–1.08)
*CD2, CD58*	rs11586238	0.24	1.13 (1.07–1.19)	0.26/0.24	1.08 (0.96–1.21)	0.26/0.26	1.05 (0.93–1.17)
*BLK*	rs2736340	0.25	1.12 (1.07–1.18)	0.27/0.25	1.10 (0.98–1.24)	0.26/0.24	1.14 (1.01–1.28)
*CD28*	rs1980422	0.24	1.12 (1.06–1.18)	0.25/0.23	1.13 (1.01–1.28)	0.26/0.23	1.15 (1.03–1.30)
*PRDM1*	rs548234	0.33	1.10 (1.05–1.16)	0.36/0.34	1.11 (1.00–1.23)	0.35/0.35	1.00 (0.90–1.10)
*CCL21*	rs2812378	0.34	1.10 (1.05–1.16)	0.38/0.34	1.17 (1.05–1.30)	0.36/0.35	1.02 (0.92–1.13)
*PTPRC*	rs10919563	0.13	0.88 (0.82–0.94)	0.11/0.13	0.82 (0.70–0.95)	0.13/0.14	0.93 (0.80–1.07)
*KIF5A, PIP4K2C*	rs1678542	0.38	0.91 (0.87–0.96)	0.34/0.37	0.86 (0.77–0.95)	0.35/0.35	0.97 (0.88–1.07)
*TRAF6*	rs540386	0.14	0.88 (0.83–0.94)	0.11/0.13	0.90 (0.77–1.05)	0.14/0.13	1.03 (0.89–1.19)
*FCGR2A*	rs12746613	0.12	1.13 (1.06–1.21)	0.14/0.12	1.17 (1.01–1.36)	0.14/0.11	1.26 (1.08–1.46)
*TAGAP*	rs394581	0.30	0.91 (0.87–0.96)	0.28/0.30	0.92 (0.82–1.03)	0.28/0.29	0.94 (0.84–1.05)
*TNFAIP3*	rs10499194	0.27	0.91 (0.87–0.96)	0.25/0.27	0.90 (0.80–1.01)	0.26/0.28	0.90 (0.81–1.00)
*IL2, IL21*	rs6822844	0.18	0.90 (0.84–0.95)	-	-	0.15/0.19	0.80 (0.71–0.91)
*IL2RA*	rs2104286	0.27	0.92 (0.87–0.97)	0.24/0.27	0.85 (0.76–0.96)	0.25/0.26	0.94 (0.84–1.04)
*IL2RB*	rs3218253	0.26	1.09 (1.03–1.15)	0.29/0.25	1.22 (1.09–1.37)	0.29/0.27	1.09 (0.98–1.22)

SNPs are ordered by significance (most significant by *P*_GWAS_ listed first); all alleles attained genome-wide significance in the published meta-analysis; Ca = Cases; Co = Controls; MAF = Minor Allele Frequency;

a = MAF in controls.

#### Comparing Model Classification Abilities

To evaluate the ability of each model to correctly classify disease status we constructed receiver operating characteristic (ROC) curves and measured the AUC; this is established methodology in determining genetic classification test efficacy [40], [41]. Higher AUCs indicate better classification. An AUC>0.5 signifies some discriminative ability; a perfect classifier has an AUC of 1. AUCs were calculated and compared using DeLong's method [42] performed within the R package, pROC [43].

#### Comparing Model Generated Risk Distributions

The risk distributions for cases and controls under each model were compared by plotting the logarithmic OR for seropositive RA for each individual ordered by risk.

#### Calculating Lifetime Risk of RA

Due to the low prevalence of RA [44], ORs approximate relative risks [45]. Therefore to calculate lifetime risks of seropositive RA we multiplied published lifetime risks by the summary OR for RA generated by our prediction models. As UK lifetime risks of RA are unknown we used estimates from a large US cohort study (2.4% for women; 1.1% for men) [46].

#### Evaluating YORA Prediction

The role of HLA, SNPs and ever-smoking status in determining age of RA onset was evaluated using individual-level OR outputs from the REGENT models in a Cox univariate analysis with gender, smoking status and smoking status-gender interaction used as covariates. Factors indicated as likely predictors of age of onset were then examined simultaneously in a multivariate analysis incorporating backward elimination of non-significant factors (*P*>0.05). We found no evidence of a “gender-smoking interaction” effect on the age of RA onset in either dataset (WTCCC *P* = 0.0823 and UKRAGG *P* = 0.8369; Table 4). This excluded a significant influence of gender on the relationship between smoking and the age at which RA developed. We therefore included both sexes when evaluating smoking's effect on the age of onset. Proportional hazards assumptions were verified using visual inspection of log-log plots [47]. To further demonstrate associations between significant factors and age of onset we constructed Kaplan-Meier estimates of the cumulative risk for cases, stratified by REGENT risk categorisation from the relevant models, alongside the presence/absence of other risk factors. We used a Cox multivariate approach to establish which four-digit *HLA-DRB1* alleles influenced age of onset (fitting all alleles simultaneously using stepwise selection, removing non-significant alleles from the final model). All time to event analyses were performed using SAS version 9.3 (SAS Institute, Cary, NC).

**Table 4:** Relationship between modelling components and age of RA onset.

	WTCCC			UKRAGG				
		Univariate Analysis^c^			Univariate Analysis		Multivariate Analysis	
Modelling Component	No. Cases Examined	*P*-Value	Hazard Ratio (95% CI)	No. Cases Examined	*P*-Value	Hazard Ratio (95% CI)	*P*-Value	Hazard ratio (95% CI)
*HLA* a *^,^* b	1022	<0.0001	1.034 (1.018–1.050)	1456	0.0004	1.025 (1.011–1.038)	0.0003	1.026 (1.012–1.040)
*SNP* a	1022	0.1804	1.043 (0.981–1.110)	284	0.294	1.075 (0.939–1.230)	-	-
*Gender* b	1021	0.2157	0.914 (0.792–1.054)	1456	0.0107	0.864 (0.722–0.967)	0.0465	0.885 (0.786–0.998)
*Smoking* b	962	0.1301	0.902 (0.789–1.031)	1361	0.0009	0.830 (0.743–0.927)	0.0041	0.848 (0.757–0.949)
*Gender-Smoking Interaction* b	961	0.0823	0.870 (0.744–1.018)	1361	0.009	0.846 (0.746–0.959)	0.8369	-

a = HLA and SNP variables represent the summary OR scores generated by the models incorporating HLA and SNP data respectively;

b = variables included in UKRAGG multivariate model after variable pruning using backwards selection and model comparison with Akaike's Information Criterion;

c = as only one parameter was significant in the WTCCC univariate analysis no multivariate model was fitted.

### Separate Analyses for ACPA-Positive RA

We undertook modelling separately for seropositive (RF and/or ACPA present) RA and ACPA-positive RA since *HLA-DRB1* allelic ORs were obtained from a meta-analysis evaluating ACPA-positive RA [35], and the shared epitope alleles, non-HLA SNPs and smoking predominantly associate with ACPA-positive disease [4], [48]–[50]. We therefore hypothesised our modelling would perform better for ACPA-positive RA. As this was confirmed in the risk categorisation results we restricted further analyses (AUC and lifetime risk calculations, examining modelling associations with age of RA onset) to ACPA-positive RA.

## Results

### Dataset Validity

#### Genetic Risk Factors

In both WTCCC and UKRAGG the effect allele frequencies and ORs for seropositive RA were generally similar to published data (Tables 2 and 3). Exceptions occurred at the four-digit *HLA-DRB1* alleles *04:08 and *15:01 (absent from controls in WTCCC and UKRAGG respectively), at *01:01, *11:01, *11:04, *13:01 and *15:01 in WTCCC and *08:01 in UKRAGG (significantly lower allele frequencies in controls than expected). The absence of *04:08 in controls was probably a chance finding since it has a frequency of 0.005. The remaining discrepancies resulted from lower four-digit tissue typing rates for these alleles in controls, which were more often typed at two-digits, compared with cases. Although this could introduce bias, especially in the context of case-control association analyses, we do not consider it significantly affected our prediction modelling because these alleles were incorporated in our models at both two-digit and four-digit resolution (in most cases in the reference meta-analysis the two-digit alleles had similar allele frequencies and ORs compared with the four-digit alleles) and our risks were obtained from an external source [35].

SNP discrepancies occurred at rs3761847 in WTCCC and rs26232 and rs540386 in UKRAGG, which had ORs in the opposite direction to published results although the dataset and meta-analysis 95% CI's overlapped for two SNPs. Additionally the minor allele frequencies (MAFs) in controls were similar to those expected. These discrepancies probably represent normal variation as opposed to systematic genotyping differences.

Most *HLA-DRB1* alleles had significant associations with RA, with only 4 (16%) alleles in WTCCC and 3 (12%) alleles in UKRAGG having 95% CIs containing 1.0. A substantial proportion of SNPs – 13 (42%) in WTCCC and 15 (48%) in UKRAGG – had 95% CIs containing 1.0 reflecting their modest effect sizes, which required large discovery cohort sizes for detection.

#### Environmental Risk Factors

The ORs for seropositive RA in ever-smokers were 3.10 (95% CI 2.22–4.37) in WTCCC and 4.32 (95% CI 3.16–5.92) in UKRAGG for males and 1.02 (95% CI 0.84–1.25) in WTCCC and 1.96 (95% CI 1.61–2.40) in UKRAGG for females. The meta-analysis gender discrepancy surrounding the effect of ever-smoking on RA risk [5] was therefore mirrored in our datasets supporting the inclusion of only males in our smoking models.

### Risk Prediction

#### Risk Categorisation

**Figure 2:**
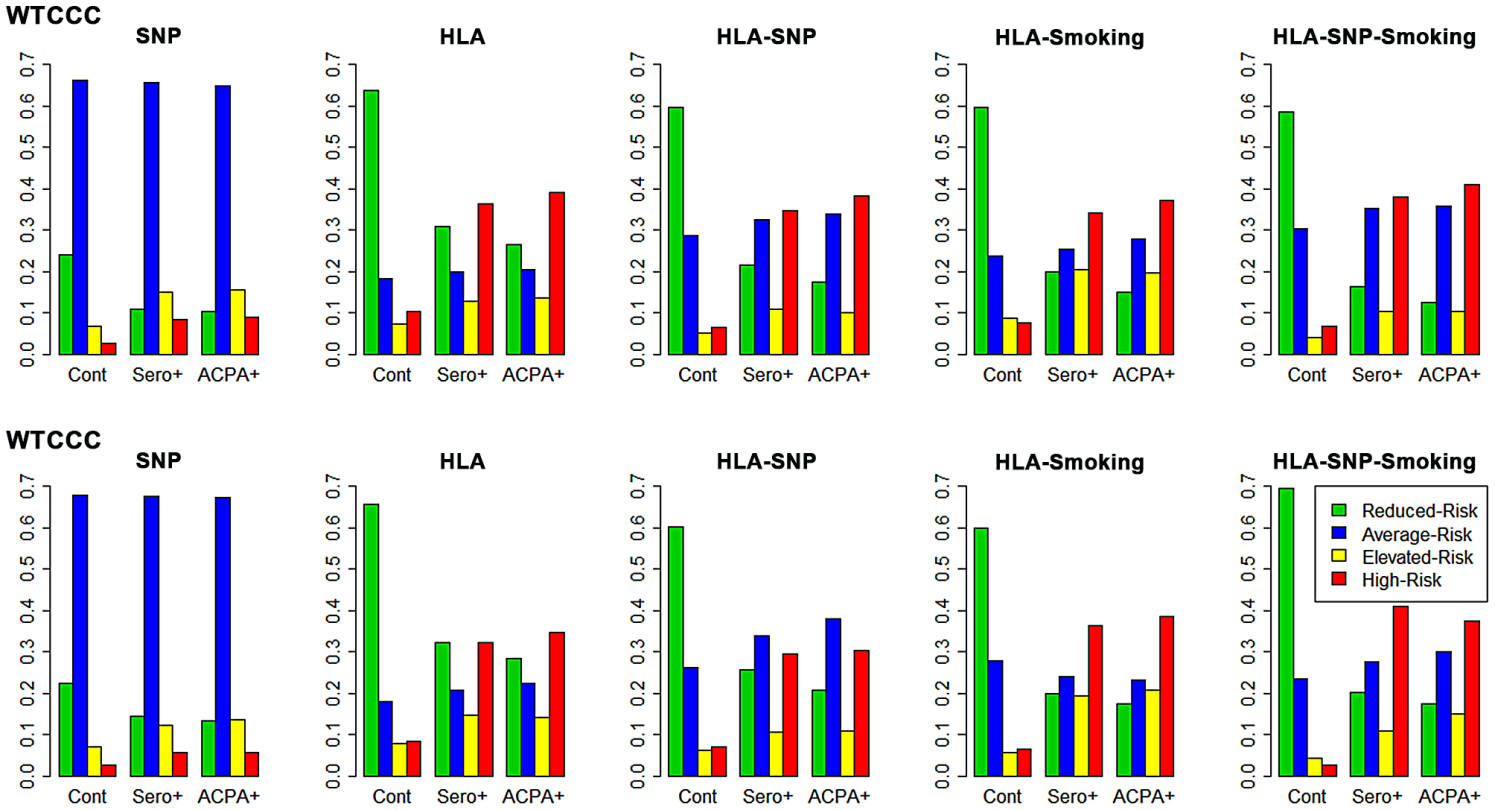
Risk categorisation of RA and controls by each prediction model. The y-axis on each graph refers to the proportion of cases/controls in each risk category; cont = controls; sero+ = seropositive RA; ACPA+ = ACPA-positive RA.

As hypothesized, our modelling more accurately categorised ACPA-positive RA as high-risk compared with seropositive RA (Figure 2 and Table S4). The HLA model provided most prediction in both datasets, classifying approximately one third of ACPA-positive RA as high-risk and two thirds of controls reduced-risk. Although the SNP model provided some prediction it classified most individuals as average-risk, reflecting the overlapping CIs generated by including many risk factors of a small effect size.

In WTCCC, the full genetic (HLA-SNP) model performed slightly better than HLA alone. Additional smoking data conferred subtle improvements in categorisation; this is particularly seen with the HLA-SNP-smoking model, which classified over half of ACPA-positive RA elevated/high-risk and 59% of controls reduced-risk.

In UKRAGG the addition of SNPs to HLA alleles increased the average-risk group size with no clear predictive benefits. The incorporation of smoking substantially improved prediction: the HLA-SNP-smoking model classified 38% ACPA-positive RA vs. 3% controls as high-risk and 70% controls vs. 18% ACPA-positive RA as reduced-risk.

The general trend of improved prediction through modelling increasing numbers of risk factors is highlighted by the ratios of the percentage of ACPA-positive cases to controls classified high-risk by each model. In WTCCC these comprise 3.4 for the SNP model, 3.8 for the HLA model, 5.8 for the HLA-SNP model, 4.8 for the HLA-smoking model and 6.0 for the HLA-SNP-smoking model. Similarly, the ratios of the percentage of controls to ACPA-positive cases classified reduced-risk in WTCCC comprise 2.3 for the SNP model, 2.4 for the HLA model, 3.4 for the HLA-SNP model, 4.0 for the HLA-smoking model and 4.7 for the HLA-SNP-smoking model. Similar findings were present in UKRAGG.

#### AUC Assessments

**Figure 3:**
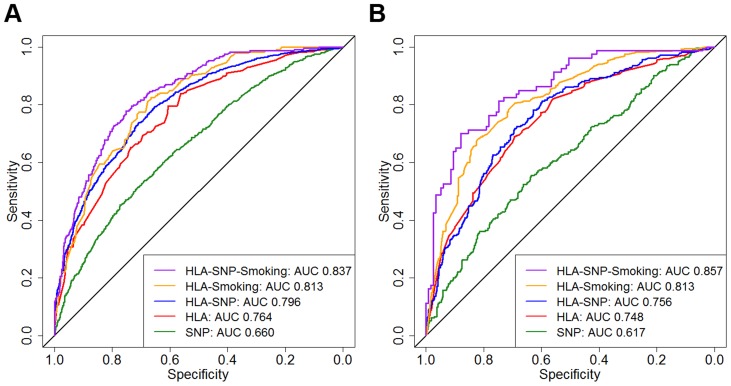
Prediction model receiver operating characteristic curves. Panel A = WTCCC; Panel B = UKRAGG; ROCs calculated for discriminating between ACPA-positive RA and controls; AUC = area under the curve. WTCCC model AUC comparisons: SNP versus HLA, *P*<0.0001; HLA versus HLA-SNP, *P* = 0.0118; HLA-SNP versus HLA-Smoking, *P* = 0.3327; HLA-Smoking versus HLA-SNP-Smoking, *P* = 0.0001. UKRAGG model AUC comparisons: SNP versus HLA, *P*<0.0001; HLA versus HLA-SNP, *P* = 0.665; HLA-SNP versus HLA-Smoking, *P* = 0.0145; HLA-Smoking versus HLA-SNP-Smoking, *P* = 0.1671.

In WTCCC AUCs for the SNP, HLA, HLA-SNP, HLA-smoking and HLA-SNP-smoking models in discriminating between ACPA-positive RA and controls comprised 0.660 (95% CI 0.638–0.681), 0.764 (95% CI 0.746–0.782), 0.796 (95% CI 0.779–0.813), 0.813 (95% CI 0.784–0.841) and 0.837 (95% CI 0.810–0.865), respectively (Figure 3). Significant differences in AUCs were observed between all three genetic models: SNP and HLA models *P*<0.0001; HLA and HLA-SNP models *P* = 0.0118. Smoking data significantly improved discrimination with differences observed between HLA and HLA-smoking model AUCs (*P* = 0.0051) and HLA-SNP and HLA-SNP-smoking model AUCs (*P* = 0.0120).

In UKRAGG AUCs for the SNP, HLA, HLA-SNP, HLA-smoking and HLA-SNP-smoking models in discriminating between ACPA-positive RA and controls comprised 0.617 (95% CI 0.577–0.656), 0.748 (95% CI 0.731–0.765), 0.756 (95% CI 0.723–0.790), 0.813 (95% CI 0.782–0.845) and 0.857 (95% CI 0.804–0.910), respectively (Figure 3). The HLA model had significantly better discrimination than the SNP model (*P*<0.0001). Combined SNP and HLA data did not improve discrimination with no differences observed between AUCs for the HLA and HLA-SNP models (*P* = 0.665) or the HLA-smoking and HLA-SNP-smoking models (*P* = 0.1671). Additional smoking information significantly improved modelling discrimination with significant differences observed between HLA and HLA-smoking model AUCs (*P* = 0.0003).

An overview of the main findings for each of the 5 prediction models, alongside the differences between them is provided in Figure S2.

#### Risk Distributions

**Figure 4:**
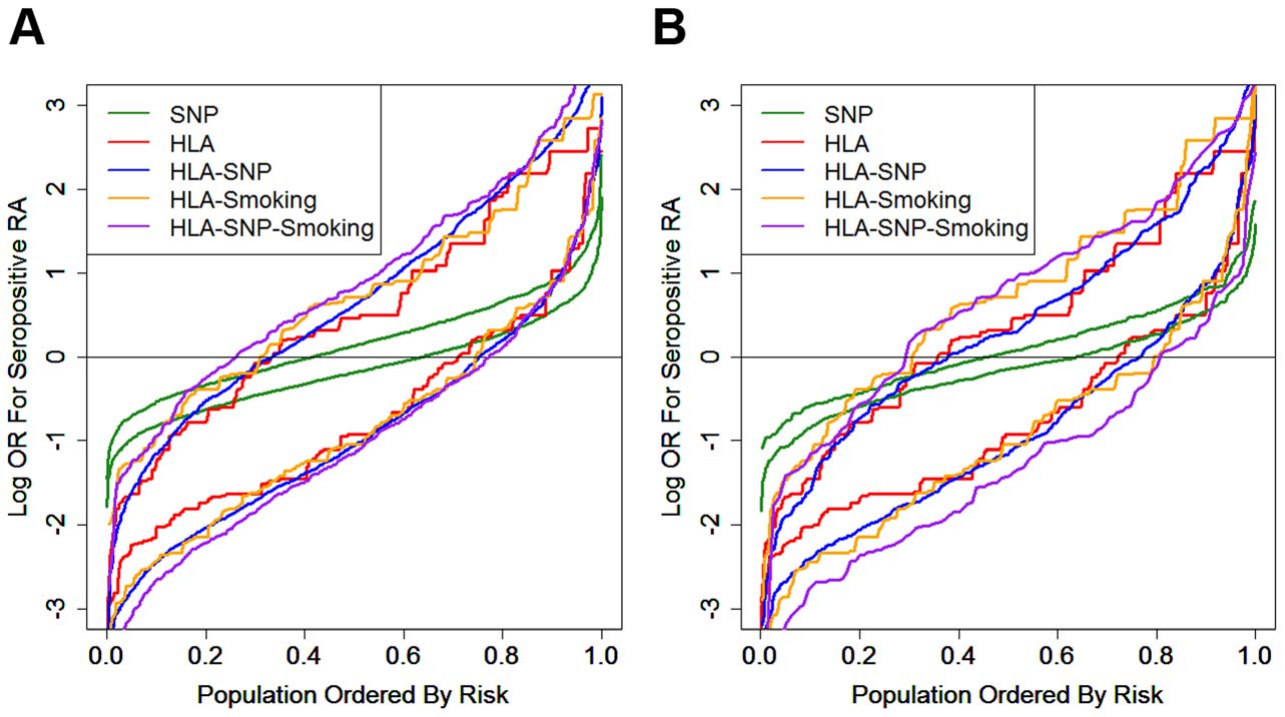
Prediction model generated risk profiles for ACPA-positive RA and controls. Panel A = WTCCC; Panel B = UKRAGG; the upper set of lines for each model refer to RA cases; the lower set of lines refer to controls; OR = odds ratio.

In both datasets the HLA model provided most risk prediction generating substantially higher and lower ORs for RA in cases and controls respectively compared with the SNP model (Figure 4).

In WTCCC the addition of other risk factors to the *HLA-DRB1* alleles resulted in further small incremental increases in ORs for RA in cases; a less pronounced reduction in risk was seen in controls.

In UKRAGG the addition of SNPs to HLA data provided no changes in case risk profiles, although a minority of controls had lower ORs. Additional smoking data resulted in significantly higher ORs for cases; only the HLA-SNP-smoking model clearly generated lower risk profiles for controls.

#### Lifetime Risk Prediction

Evaluating risks using genetics (HLA-SNP model) alone the highest risk WTCCC ACPA-positive case had an OR for seropositive RA of 79; as a male his lifetime risk was estimated at 86%. The highest risk control had an OR of 22; as a female her lifetime risk was estimated at 53%. Despite such high individual odds only a relative minority had relevant increased lifetime risks: using the same HLA-SNP model 49 (4.61%) ACPA-positive cases and 1 (0.07%) control had ORs for seropositive RA>20 (lifetime risks >48% if female and >22% if male) in WTCCC. In UKRAGG 9 (3.06%) ACPA-positive cases and 1 (0.17%) control had ORs>20.

The HLA-SNP-smoking model identified the greatest proportion of cases with substantially increased lifetime risks for RA. This model identified 18 (7.53%) and 3 (3.75%) ACPA-positive male cases to have ORs for seropositive RA>20 (lifetime risk >22%) in WTCCC and UKRAGG respectively; no controls had ORs>20.

### Younger Onset RA Prediction

In WTCCC the HLA model summary OR score was the only significant predictor of age of RA onset (Table 4). The hazard ratio (HR) was 1.034 (*P*<0.0001), which indicated that the hazard (the rate at which RA occurred) was greater in individuals with higher HLA derived ORs than those with lower ORs. Therefore a higher HLA model generated risk score associated with RA occurring at a faster rate and thus YORA. Conversely ever-smoking was associated with older onset RA: the HR of 0.902 indicated a smaller hazard (RA occurred at a slower rate) in ever-smokers compared with never-smokers, although this was not significant (*P* = 0.1301).

In UKRAGG the HLA model summary OR score, gender and smoking status were significant independent predictors of age of onset. An increasing HLA summary OR score associated with YORA (*P* = 0.0003, HR 1.026); ever-smoking (*P* = 0.0041, HR 0.848) and male gender (*P* = 0.0465, HR 0.885) associated with older onset RA.

We considered that the non-significant relationship between smoking and age of onset in WTCCC reflected a limited sample size with our power to detect a 0.88 HR in the 962 WTCCC cases approximately 51% compared with 65% for the 1,361 UKRAGG cases. We therefore undertook a pooled analysis of both datasets (incorporating an additional “study” variable to account for dataset median age of onset differences). This confirmed that HLA derived risk scores significantly associated with YORA (*P*<0.0001, HR 1.030) and ever-smoking significantly associated with older onset RA (*P* = 0.0489, HR 0.889).

**Figure 5:**
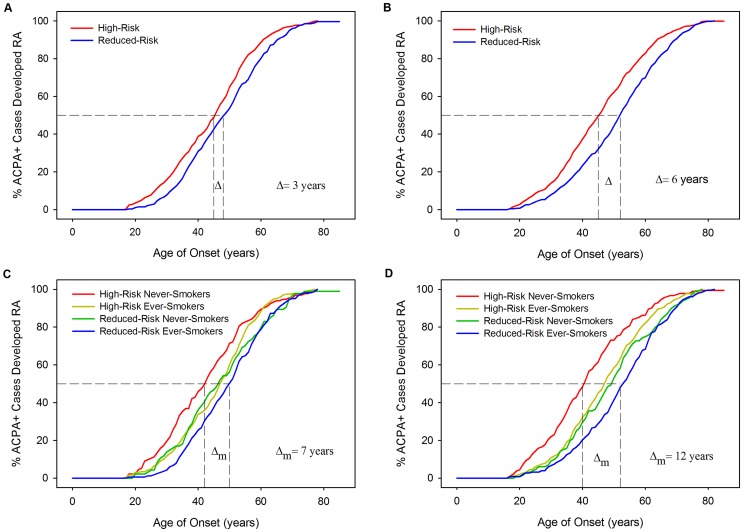
Kaplan-Meier curves: RA age of onset stratified by HLA model risk categorisation and smoking status. Panel A = WTCCC Curves Stratified By Risk Categorisation; Panel B = UKRAGG Curves Stratified By Risk Categorisation; Panel C = WTCCC Curves Stratified By Risk Categorisation and Ever-Smoking Status; Panel D = UKRAGG Curves Stratified By Risk Categorisation and Ever-Smoking Status; Δ = change in onset age; Δ_m_ = maximum change in onset age across strata.

Kaplan-Meier curves of age of onset stratified by HLA model risk categorisation further demonstrate the association of HLA risk profiles with YORA (Figure 5) with cases classified high-risk having significantly younger onset ages compared to those classified reduced-risk. In WTCCC the difference in the median time to RA (time point at which half the cases have developed RA) was 3 years between those classed high- and reduced-risk (Log-Rank = 11.43; *P* = 0.0007). In UKRAGG a stronger association was seen (Log-Rank = 27.33; *P*<0.0001) with a difference in median time to RA onset between risk groups of 6 years. Further stratification by ever-smoking status demonstrated a trend towards an older onset age in ever-smokers. In WTCCC the median time to onset difference between high-risk never-smokers and reduced-risk ever-smokers was 7 years (Log-Rank = 14.42; *P* = 0.0024); a larger disparity was seen in UKRAGG with a difference of 12 years observed (Log-Rank = 46.2505; *P*<0.0001).

Examining which four-digit resolution *HLA-DRB1* alleles influenced onset age revealed significant associations between age of onset and *03:01 (*P* = 0.0313), *04:01 (*P* = 0.0001), *04:08 (*P* = 0.0032) and *13:02 (*P* = 0.0097) in WTCCC and *04:01 (*P*<0.0001) and *04:04 (*P* = 0.0243) in UKRAGG. Three of these alleles (*04:01, *04:04 and *04:08) are shared-epitope alleles.

## Discussion

We have demonstrated that predicting RA development is possible with our prediction models able to identify individuals with clinically relevant increased risks for seropositive RA. Our modelling indicates that most prediction is provided by *HLA-DRB1* alleles and, to a lesser extent, smoking in males; non-HLA susceptibility SNPs provide only minor predictive benefits. These findings are consistent with the estimations of heritability variance conferred by different genetic components. We have also shown it is possible to predict the age of RA onset, using information on HLA and smoking to identify those at risk of younger and older onset RA, respectively. Whilst our novel modelling approach, which uses computer simulation-based categorisation alongside a greater number of HLA alleles, significantly improves upon the discriminative abilities of existing models [26], [27] it remains unsuitable for population screening with only a minority at significantly increased lifetime risks for RA.

Our approach provides some potential advantages over existing RA prediction modelling [26], [27]. Firstly, by using a simulated population to generate risk profiles we do not require an entire population of real-life data to stratify risks. In contrast existing approaches categorise wGRS scores using their Gaussian distribution in control groups. Secondly, our CI-based approach considers the precision with which risk factor effect sizes are known when classifying risk; this prevents classifying people high-risk if their risk is imprecisely known. Thirdly, our models provide greater discrimination: the highest AUC for existing clinical-genetic models in discerning ACPA-positive RA from controls is 0.752; the highest AUC for our clinical-genetic model is 0.857.

SNPs provided only minor improvements in prediction, highlighting the limitations of genome-wide association study (GWAS) derived data in this field. Although GWAS-established SNPs have helped identify cellular pathways relevant to RA pathogenesis [51] their modest effect sizes limit their predictive utility. It has been proposed that the missing heritability of RA may reflect the involvement of rare variants of large effect sizes or structural variants [52]. Alternative genotyping technologies such as next-generation sequencing may identify these variants, although only loci with large effect sizes will substantially improve prediction modelling.

Although individuals with clinically relevant increased lifetime risks (such as 86%) for RA were identified there was, overall, only a minority of individuals at a significantly elevated risk: 7% of ACPA-positive individuals had lifetime risks of 22% or more when evaluated using all available risk factors. Therefore despite high AUCs our modelling is unsuitable for population level screening. However, if its use was targeted to groups with *a priori* increased risks, such as first degree relatives of RA probands [53]–[55], then a substantially greater proportion of very high-risk individuals might be identified.

Individuals classified high-risk by our HLA model were more likely to develop RA at a younger age. This finding – mainly attributable to the *04:01 allele – is supported by existing literature. Hellier *et al* reported a higher frequency of *04 RA associated alleles in YORA (present in 52% of 262 RA cases with onset age <60) compared with elderly onset RA (present in 37% of 60 cases with onset age >60; *P* = 0.045) [18]. Similarly, Wu *et al* identified a significantly younger age of onset in Caucasian RA patients carrying shared epitope encoding *04 alleles (*P* = 0.0003) [19]. Other studies report positive correlations between YORA and shared epitope alleles [25], [56]. Our finding of ever-smoking associating with older onset RA is less established. It has only been examined in three relatively small studies, with contrasting outcomes: one study reported a significant relationship between smoking at disease onset and a younger onset age [57]; one reported a younger onset age in current vs. never-smokers (although ex-smokers had older onset RA in comparison to both these groups) [58]; the final study found no association [59]. Our findings – demonstrated in 2,323 individuals across two independent datasets – are biologically plausible. As risk genotypes are present from birth they can exert their effects on disease risk throughout an individual's lifetime; therefore possessing high-risk *HLA-DRB1* alleles predisposes to RA at a younger age. In contrast the risk of RA increases as more cigarettes are smoked [60], [61] and smokers are exposed to more cigarettes as they age; therefore smokers are more likely to develop RA as they get older because they have been exposed to more cigarettes and thus smoking associates with older onset RA. This logic also explains why ever-smoking associates with older onset RA in both men and women, with heavy smoking a risk factor for RA in both genders [5]. We were, however, unable to incorporate heavy smoking in our prediction modelling due to a paucity of data on smoking pack-years in WTCCC/UKRAGG.

We incorporated many genetic risk factors in our modelling but included only one environmental risk factor, smoking. This reflects uncertainty regarding relevant environmental risks alongside limited environmental data within current genetic datasets. Although many environmental factors are linked to RA their associations are usually identified in case-control studies, which are subject to multiple biases, rather than cohort studies. Examples include alcohol consumption [36], parity [62], [63] and oral contraceptive pill use [64]. Better characterisation of environmental risks will enhance predictive modelling.

Our modelling has several limitations. Firstly, WTCCC participants were included in the meta-analyses that we obtained our genetic risk loci data from; however WTCCC comprised only a proportion of the meta-analyses datasets (20% of the HLA meta-analysis; 29% of the SNP meta-analysis) and our findings were independently replicated in UKRAGG. Secondly, missing data meant the number of individuals included in each model fell as more risk factors were included; this is particularly seen in models incorporating smoking. Thirdly, due to marked heterogeneity in published data on gene-gene/gene-environment interactions we assumed independence between these factors despite known interactions existing between the shared epitope alleles and *PTPN22* and smoking [6], [7], [29], [37], [38].

Improving RA prediction requires better clarification of its genetic and environmental risk factors. Identifying risk factors with large effect sizes of known precision will most enhance prediction modelling. This could be facilitated through fine-mapping studies that better tag causal variants [65] alongside prospective cohort studies examining environmental risk factors in RA cases subdivided by ACPA status, with increasing evidence that risks differ between these serological subsets [36], [66]. It is, however, unlikely that identifying such risk factors will substantially increase the proportion of individuals with clinically relevant high disease risks. We therefore consider that prediction modelling requires evaluation in *a priori* higher risk groups. In this context it may identify sufficient numbers of very high-risk individuals, facilitating a better understanding of pre-RA immunopathology and enabling the assessment of primary prevention strategies.

## Supporting Information

Figure S1 REGENT stage 1- simulation of general population risk profiles: example using a model incorporating 3 SNPs.
(TIFF)

Figure S2 Main findings of each prediction model.
(TIFF)

Table S1 Risk categorisation results for models incorporating smoking in females. Data are number (%) unless stated otherwise; ^a^ = AUCs calculated using ACPA-positive cases.
(DOCX)

Table S2 Proxy SNPs used in modelling. a = proxy SNP obtained using 1,000 Genomes CEU population panel [39]; b = proxy SNP obtained using HapMap release 22 CEU population panel [39]; c = proxy SNP obtained using Ricopili (Broad Institute, Boston, USA) from the GWAS meta-analysis of RA risk (http://www.broadinstitute.org/mpg/ricopili/).
(DOCX)

Table S3 Two-digit, four-digit and mixed-digit hla prediction model results, showing similar discriminative abilities. Data are number (%) unless stated otherwise; Sero+ = seropositive RA; ACPA+ = ACPA-positive RA; AUCs calculated using ACPA-positive cases; two-digit model evaluated individuals with all available HLA data collapsed down to two-digit resolution.
(DOCX)

Table S4 Prediction model results: risk categorisation. Data are number (%); Sero+ = seropositive RA; ACPA+ = ACPA-positive RA.
(DOCX)
