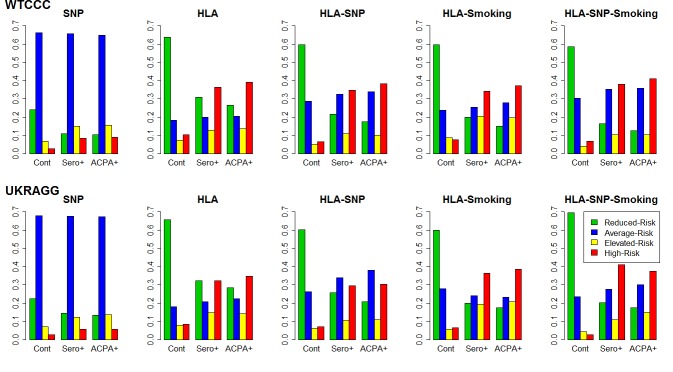# Correction: Predicting the Risk of Rheumatoid Arthritis and Its Age of Onset through Modelling Genetic Risk Variants with Smoking

**DOI:** 10.1371/annotation/a86ab397-f58c-4144-a241-4957836a728a

**Published:** 2013-10-18

**Authors:** Ian C. Scott, Seth D. Seegobin, Sophia Steer, Rachael Tan, Paola Forabosco, Anne Hinks, Stephen Eyre, Ann W. Morgan, Anthony G. Wilson, Lynne J. Hocking, Paul Wordsworth, Anne Barton, Jane Worthington, Andrew P. Cope, Cathryn M. Lewis

The bottom set of graphs in Figure 2 was labelled incorrectly. It should read UKRAGG rather than WTCCC. Please download the correct file here: 

**Figure:**