# Supplementary figures and images for: Predicting the Risk of Rheumatoid Arthritis and Its Age of Onset through Modelling Genetic Risk Variants with Smoking
Source: PLoS Genet. 2013 Sep 19;9(9):e1003808. doi: 10.1371/journal.pgen.1003808 (PMC3778023; doi:10.1371/journal.pgen.1003808)

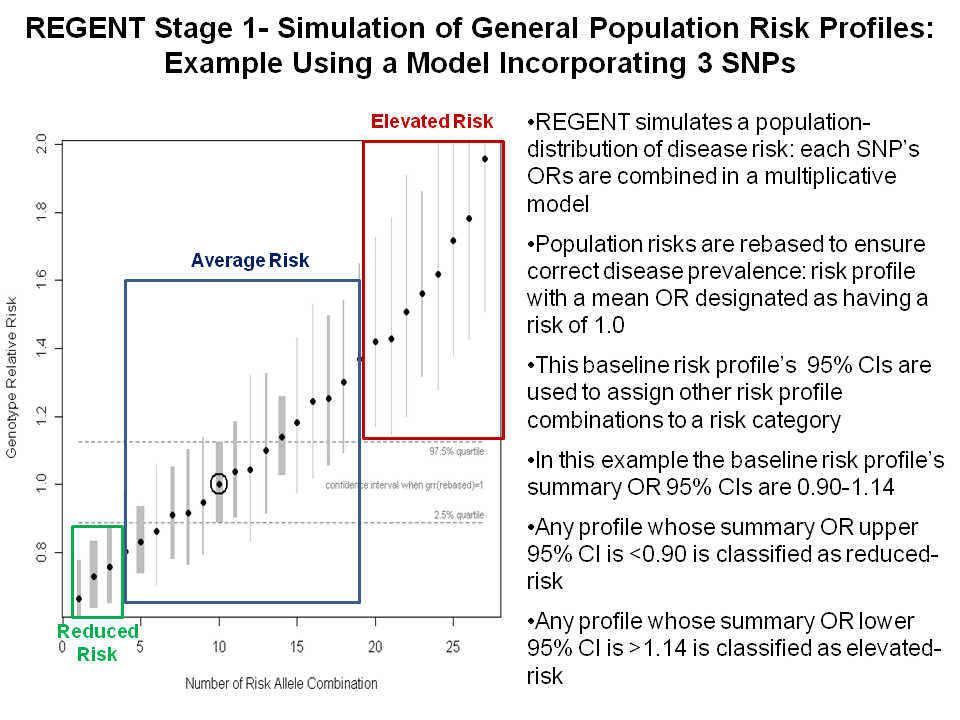

Supplement: Figure S1 — REGENT stage 1- simulation of general population risk profiles: example using a model incorporating 3 SNPs. (TIFF) [file pgen.1003808.s001.tiff]
