# Supplementary material for: Predicting the Risk of Rheumatoid Arthritis and Its Age of Onset through Modelling Genetic Risk Variants with Smoking
Source: PLoS Genet. 2013 Sep 19;9(9):e1003808. doi: 10.1371/journal.pgen.1003808 (PMC3778023; doi:10.1371/journal.pgen.1003808)
Supplement: Table S1 — Risk categorisation results for models incorporating smoking in females. Data are number (%) unless stated otherwise; a = AUCs calculated using ACPA-positive cases. (DOCX) [file pgen.1003808.s003.docx]

**Table S1. Risk Categorisation Results for Models Incorporating Smoking in Females**

| *Risk Category* | **WTCCC HLA-Smoking Model** | | | **UKRAGG HLA-Smoking Model** | | | **WTCCC HLA-SNP-Smoking Model** | | | **UKRAGG HLA-SNP-Smoking Model** | | |
| --- | --- | --- | --- | --- | --- | --- | --- | --- | --- | --- | --- | --- |
|  | Sero+ n=947 | ACPA+ n=751 | Controls n=736 | Sero+ n= 1355 | ACPA+ n=983 | Controls n=606 | Sero+ n=947 | ACPA+ n=751 | Controls n=736 | Sero+ n= 333 | ACPA+ n=182 | Controls n=231 |
| *Reduced* | 268 (28.3) | 179 (23.8) | 437 (59.4) | 417 (30.8) | 264 (26.9) | 387 (63.9) | 202 (21.3) | 127 (16.9) | 401 (54.5) | 84 (25.2) | 38 (20.9) | 137 (59.3) |
| *Average* | 330 (34.8) | 269 (35.8) | 225 (30.6) | 502 (37.0) | 369 (37.5) | 159 (26.2) | 336 (35.5) | 273 (36.4) | 254 (34.5) | 129 (38.7) | 81 (44.5) | 63 (27.3) |
| *Elevated* | 19 (2.0) | 17 (2.3) | 6 (0.8) | 35 (2.6) | 25 (2.5) | 10 (1.7) | 107 (11.3) | 88 (11.7) | 32 (4.3) | 30 (9.0) | 16 (8.8) | 14 (6.1) |
| *High* | 330 (34.8) | 286 (38.1) | 68 (9.2) | 401 (29.6) | 325 (33.1) | 50 (8.3) | 302 (31.9) | 263 (35.0) | 49 (6.7) | 90 (27.0) | 47 (25.8) | 17 (7.4) |
| *AUC*^a^  *(95% CI)* | 0.758 (0.733-0.782) | | | 0.749 (0.724-0.773) | | | 0.783 (0.760-0.806) | | | 0.738 (0.690-0.786) | | |

Data are number (%) unless stated otherwise; ^a^ = AUCs calculated using ACPA-positive cases.
