# Supplementary material for: Predicting the Risk of Rheumatoid Arthritis and Its Age of Onset through Modelling Genetic Risk Variants with Smoking
Source: PLoS Genet. 2013 Sep 19;9(9):e1003808. doi: 10.1371/journal.pgen.1003808 (PMC3778023; doi:10.1371/journal.pgen.1003808)
Supplement: Table S2 — Proxy SNPs used in modelling. a = proxy SNP obtained using 1,000 Genomes CEU population panel [39]; b = proxy SNP obtained using HapMap release 22 CEU population panel [39]; c = proxy SNP obtained using Ricopili (Broad Institute, Boston, USA) from the GWAS meta-analysis of RA risk (http://www.broadinstitute.org/mpg/ricopili/). (DOCX) [file pgen.1003808.s004.docx]

**Table S2. Proxy SNPs Used In Modelling**

| **WTCCC** | | | |
| --- | --- | --- | --- |
| **Meta-Analysis SNP [**[**34**](#_ENREF_34)**]** | **Loci** | **Proxy SNP Used** | **R^2^** |
| rs10499194 | *TNFAIP3* | rs13207033^a^ | 1 |
| rs10488631 | *IRF5* | rs12531711^a^ | 1 |
| rs3761847 | *TRAF1, C5* | rs10118357^a^ | 0.967 |
| rs10865035 | *AFF3* | rs9653442^b^ | 0.967 |
| rs26232 | *C5orf30* | rs35797^b^ | 0.901 |
| rs3093023 | *CCR6* | rs6907666^b^ | 0.933 |
| rs706778 | *IL2RA* | rs10795791^b^ | 0.934 |
| rs5029937 | *TNFAIP3* | rs5029939^b^ | 1 |
| rs2476601 | *PTPN22* | rs6679677^b^ | 1 |
| rs13031237 | *REL* | rs702873^c^ | 1 |
| rs7574865 | *STAT4* | rs3821236^c^ | 1 |
| **UKRAGG** | | | |
| **Meta-Analysis SNP [**[**34**](#_ENREF_34)**]** | **Loci** | **Proxy SNP Used** | **R^2^** |
| rs10499194 | *TNFAIP3* | rs12527282^a^ | 1 |
| rs3890745 | *TNFRSF14* | rs10910099^a^ | 0.926 |

a = proxy SNP obtained using 1,000 Genomes CEU population panel [[39](#_ENREF_39)]; b = proxy SNP obtained using HapMap release 22 CEU population panel [[39](#_ENREF_39)]; c = proxy SNP obtained using Ricopili (Broad Institute, Boston, USA) from the GWAS meta-analysis of RA risk (http://www.broadinstitute.org/mpg/ricopili/).
