# Supplementary material for: Predicting the Risk of Rheumatoid Arthritis and Its Age of Onset through Modelling Genetic Risk Variants with Smoking
Source: PLoS Genet. 2013 Sep 19;9(9):e1003808. doi: 10.1371/journal.pgen.1003808 (PMC3778023; doi:10.1371/journal.pgen.1003808)
Supplement: Table S3 — Two-digit, four-digit and mixed-digit hla prediction model results, showing similar discriminative abilities. Data are number (%) unless stated otherwise; Sero+ = seropositive RA; ACPA+ = ACPA-positive RA; AUCs calculated using ACPA-positive cases; two-digit model evaluated individuals with all available HLA data collapsed down to two-digit resolution. (DOCX) [file pgen.1003808.s005.docx]

**Table S3. Two-Digit, Four-Digit and Mixed-Digit HLA Prediction Model Results, Showing Similar Discriminative Abilities**

| **WTCCC** | | | | | | | | | |
| --- | --- | --- | --- | --- | --- | --- | --- | --- | --- |
| *Risk Category* | **Two-Digit Resolution Model** | | | **Four-Digit Resolution Model** | | | **Mixed-Digit Resolution Model** | | |
|  | Sero+ n=1516 | ACPA+ n=1061 | Controls n=1647 | Sero+ n=1342 | ACPA+ n=966 | Controls n=1126 | Sero+ n=1516 | ACPA+ n=1061 | Controls n=1647 |
| *Reduced* | 440 (29.0) | 267 (25.2) | 973 (59.1) | 386 (28.8) | 242 (25.1) | 678 (60.2) | 471 (31.1) | 283 (26.7) | 1052 (63.9) |
| *Average* | 108 (7.1) | 78 (7.4) | 140 (8.5) | 135 (10.1) | 102 (10.6) | 132 (11.7) | 301 (19.9) | 218 (20.5) | 304 (18.5) |
| *Elevated* | 406 (26.8) | 296 (27.9) | 340 (20.6) | 180 (13.4) | 141 (14.6) | 124 (11.0) | 194 (12.8) | 145 (13.7) | 121 (7.3) |
| *High* | 562 (37.1) | 420 (39.6) | 194 (11.8) | 641 (47.8) | 481 (49.8) | 192 (17.1) | 550 (36.3) | 415 (39.1) | 170 (10.3) |
| *AUC (95% CI)* | 0.744 (0.726-0.763) | | | 0.765 (0.745-0.785) | | | 0.764 (0.746-0.782) | | |
| **UKRAGG** | | | | | | | | | |
| *Risk Category* | **Two-Digit Resolution Model** | | | **Four-Digit Resolution Model** | | | **Mixed-Digit Resolution Model** | | |
|  | Sero+ n=2623 | ACPA+ n=1508 | Controls n=1500 | Sero+ n=1534 | ACPA+ n=1108 | Controls n=735 | Sero+ n=2623 | ACPA+ n=1508 | Controls n=1500 |
| *Reduced* | 802 (30.6) | 409 (27.1) | 931 (62.1) | 342 (22.3) | 231 (20.8) | 393 (53.5) | 844 (32.2) | 430 (28.5) | 987 (65.8) |
| *Average* | 186 (7.1) | 126 (8.4) | 138 (9.2) | 157 (10.2) | 115 (10.4) | 99 (13.5) | 544 (20.7) | 337 (22.3) | 270 (18.0) |
| *Elevated* | 732 (27.9) | 409 (27.1) | 288 (19.2) | 200 (13.0) | 151 (13.6) | 82 (11.2) | 388 (14.8) | 215 (14.3) | 118 (7.9) |
| *High* | 903 (34.4) | 564 (37.4) | 143 (9.5) | 835 (54.4) | 611 (55.1) | 161 (21.9) | 847 (32.3) | 526 (34.9) | 125 (8.3) |
| *AUC (95% CI)* | 0.743 (0.725-0.760) | | | 0.743 (0.720-0.766) | | | 0.748 (0.731-0.765) | | |

Data are number (%) unless stated otherwise; Sero+ = seropositive RA; ACPA+ = ACPA-positive RA; AUCs calculated using ACPA-positive cases; two-digit model evaluated individuals with all available HLA data collapsed down to two-digit resolution.
