# Supplementary material for: Predicting the Risk of Rheumatoid Arthritis and Its Age of Onset through Modelling Genetic Risk Variants with Smoking
Source: PLoS Genet. 2013 Sep 19;9(9):e1003808. doi: 10.1371/journal.pgen.1003808 (PMC3778023; doi:10.1371/journal.pgen.1003808)
Supplement: Table S4 — Prediction model results: risk categorisation. Data are number (%); Sero+ = seropositive RA; ACPA+ = ACPA-positive RA. (DOCX) [file pgen.1003808.s006.docx]

**Table S4. Prediction Model Results: Risk Categorisation**

| **WTCCC Prediction Models** | | | | | | | | | | | | | | | |
| --- | --- | --- | --- | --- | --- | --- | --- | --- | --- | --- | --- | --- | --- | --- | --- |
| *Risk Category* | **HLA Model** | | | **SNP Model** | | | **HLA-SNP Model** | | | **HLA-Smoking Model** | | | **HLA-SNP-Smoking Model** | | |
|  | Sero+ n=1516 | ACPA+ n=1061 | Controls n=1647 | Sero+ n= 1516 | ACPA+ n=1061 | Controls n=1476 | Sero+ n=1516 | ACPA+ n=1061 | Controls n=1476 | Sero+ n= 287 | ACPA+ n=239 | Controls n=739 | Sero+ n= 287 | ACPA+ n=239 | Controls n=739 |
| *Reduced* | 471 (31.1) | 283 (26.7) | 1052 (63.9) | 167 (11.0) | 109 (10.3) | 355 (24.1) | 329 (21.7) | 186 (17.5) | 880 (59.6) | 57 (19.9) | 36 (15.1) | 442 (59.8) | 47 (16.4) | 30 (12.6) | 434 (58.7) |
| *Average* | 301 (19.9) | 218 (20.5) | 304 (18.5) | 996 (65.7) | 690 (65.0) | 979 (66.3) | 494 (32.6) | 361 (34.0) | 423 (28.7) | 73 (25.4) | 67 (28.0) | 176 (23.8) | 101 (35.2) | 86 (36.0) | 225 (30.4) |
| *Elevated* | 194 (12.8) | 145 (13.7) | 121 (7.3) | 226 (14.9) | 165 (15.6) | 102 (6.9) | 167 (11.0) | 108 (10.2) | 75 (5.1) | 59 (20.6) | 47 (19.7) | 64 (8.7) | 30 (10.5) | 25 (10.5) | 30 (4.1) |
| *High* | 550 (36.3) | 415 (39.1) | 170 (10.3) | 127 (8.4) | 97 (9.1) | 40 (2.7) | 526 (34.7) | 406 (38.3) | 98 (6.6) | 98 (34.1) | 89 (37.2) | 57 (7.7) | 109 (38.0) | 98 (41.0) | 50 (6.8) |
| **UKRAGG Prediction Models** | | | | | | | | | | | | | | | |
| *Risk Category* | **HLA Model** | | | **SNP Model** | | | **HLA-SNP Model** | | | **HLA-Smoking Model** | | | **HLA-SNP-Smoking Model** | | |
|  | Sero+ n=2623 | ACPA+ n=1508 | Controls n=1500 | Sero+ n=937 | ACPA+ n=294 | Controls n=573 | Sero+ n=937 | ACPA+ n=294 | Controls n=573 | Sero+ n=529 | ACPA+ n=413 | Controls n=322 | Sero+ n=119 | ACPA+ n=80 | Controls n=115 |
| *Reduced* | 844 (32.2) | 430 (28.5) | 987 (65.8) | 135 (14.4) | 39 (13.3) | 128 (22.3) | 242 (25.8) | 61 (20.7) | 346 (60.4) | 106 (20.0) | 72 (17.4) | 193 (59.9) | 24 (20.2) | 14 (17.5) | 80 (69.6) |
| *Average* | 544 (20.7) | 337 (22.3) | 270 (18.0) | 633 (67.6) | 198 (67.3) | 390 (68.1) | 319 (34.0) | 112 (38.1) | 150 (26.2) | 127 (24.0) | 96 (23.2) | 90 (28.0) | 33 (27.7) | 24 (30.0) | 27 (23.5) |
| *Elevated* | 388 (14.8) | 215 (14.3) | 118 (7.9) | 116 (12.4) | 40 (13.6) | 40 (7.0) | 99 (10.6) | 32 (10.9) | 36 (6.3) | 103 (19.5) | 86 (20.8) | 18 (5.6) | 13 (10.9) | 12 (15.0) | 5 (4.3) |
| *High* | 847 (32.3) | 526 (34.9) | 125 (8.3) | 53 (5.7) | 17 (5.8) | 15 (2.6) | 277 (29.6) | 89 (30.3) | 41 (7.2) | 193 (36.5) | 159 (38.5) | 21 (6.5) | 49 (41.2) | 30 (37.5) | 3 (2.6) |

Data are number (%); Sero+ = seropositive RA; ACPA+ = ACPA-positive RA.
